# Comparison of efficacy of long follicular phase regimen and antagonist regimen on pregnancy outcome of fresh cycle or freeze-thawed cycle embryo transfer

**DOI:** 10.12669/pjms.40.10.9050

**Published:** 2024-11

**Authors:** Pengtao Li, Jiawei Zhai, Ting Liu, Mengyuan Guo, Yuzhen Wang

**Affiliations:** 1 Pengtao Li, Department of Reproductive Medicine, Baoding Maternal and Child Health Care Hospital, Baoding 071000, Hebei, China; 2 Jiawei Zhai, Department of Reproductive Medicine, Baoding Maternal and Child Health Care Hospital, Baoding 071000, Hebei, China; 3 Ting Liu, Department of Reproductive Medicine, Baoding Maternal and Child Health Care Hospital, Baoding 071000, Hebei, China; 4 Mengyuan Guo, Department of Reproductive Medicine, Baoding Maternal and Child Health Care Hospital, Baoding 071000, Hebei, China; 5 Yuzhen Wang, Department of Reproductive Medicine, Baoding Maternal and Child Health Care Hospital, Baoding 071000, Hebei, China

**Keywords:** Long follicular phase regimen, antagonist regimen, freeze-thawed cycle transfer, Fresh cycle transfer

## Abstract

**Objective:**

To compare the pregnancy outcome of the fresh cycle or freeze-thaw cycle embryo transfer of patients treated with a long follicular phase regimen and antagonist regimen, and explore the clinical therapeutic effect of the two regimens.

**Methods:**

This was a retrospective study. The data of a total of 543 patients who underwent in vitro fertilization/intracytoplasmic sperm injection and embryo transfer (IVF/ICSI-ET) or frozen-thawed embryo transfer (FET) in Baoding Maternal and Child Health Care Hospital from January 2020 to December 2022 were retrospectively analyzed in this study and were divided into four group to analyze the basic conditions, medication, laboratory indicators and clinical outcomes after embryo transfer of patients in each group.

**Results:**

The pregnancy rate and implantation rate in Groups A, B and C were higher than those in Group-D, and the differences were statistically significant (*p<*0.05). The difference in multiple pregnancy rate and abortion rate among groups was not statistically significant (*p>*0.05). The influencing factors of clinical pregnancy rate were identified by binary Logistic regression analysis. Advanced age was found to be a risk factor for improving the pregnancy rate, while the increase in the number of oocytes retrieved is a protective factor for improving the pregnancy rate. The differences were statistically significant (*p<*0.05).

**Conclusion:**

The antagonist regimen has a low dosage and short medication time and can achieve a high embryo utilization rate and blastula formation rate, saving time and cost for patients.

## INTRODUCTION

Controlled hyperovulation is an important part of in vitro fertilization-embryo transfer (IVF-ET), and is the key link to achieving pregnancy for patients receiving treatment. Every reproductive clinician develops reasonable, effective and safe ovulation-inducing regimens according to patients’ own conditions. At present, the clinically common regimens include long luteal phase gonadotropin releasing hormone agonist (GnRH-a) regimen, long follicular phase GnRH-a regimen (hereinafter referred to as long follicular phase regimen), short GnRH-a regimen, ultra-long GnRH-a regimen, minimal stimulation regimen, natural cycle regimen and antagonist regimen, each of which is applicable to different groups. Each regimen has its own advantages. For instance, the long follicular phase GnRH-a regimen can achieve a greater number of oocytes, more transferable embryos, better endometrial receptivity and a lower cycle cancellation rate.^1^,^2^ The antagonist regimen has attracted much attention due to its advantages such as low dosage, short treatment period, low incidence of ovarian hyperstimulation, and equivalent or even better success rate than traditional agonist regimen.^3^,^5^

In this study, the application of the long follicular phase GnRH-a regimen and the antagonist regimen in our hospital were retrospectively analyzed. In addition, the number of oocytes retrieved, embryo utilization rate, blastula formation rate, implantation rate, abortion rate, multiple pregnancy rate and other indicators of patients after receiving ovulation induction by the two regimens were compared to explore the effects of the two regimens to provide a reference for the clinical work in the future, and to analyze which option is safer and more effective.

## METHODS

This was a retrospective study. The data of a total of 543 patients who received IVF/ICSI-ET or FET in the Reproductive Medicine Department of Baoding Maternal and Child Health Care Hospital from January 2020 to December 2022 were retrospectively analyzed, including 92 cycles of freeze-thawed cycle transfer by long follicular phase regimen, 239 cycles of fresh cycle transfer by long follicular phase regimen, 55 cycles of freeze-thawed cycle transfer by antagonist regimen and 157 cycles of fresh cycle transfer by antagonist regimen.

### Ethical Approval:

The study was approved by the Institutional Ethics Committee of Baoding Maternal and Child Health Care Hospital (No.:2023-01-K005; date: May 12,2023), and written informed consent was obtained from all participants.

### Inclusion criteria:

- Patients who received ovulation induction by long follicular phase regimen or antagonist regimen and fresh embryo transfer or first freeze-thawed embryo transfer after whole embryo freezing;
- Female patients aged 25~40;
- Anti-Mullerian hormone (AMH)≥1 ng/ml;
- Partners without any genetic disease.

### Exclusion criteria:

- Patients with uterine malformation;
- Patients with endometriosis;
- Patients with the polycystic ovarian syndrome.

The long-acting GnRH-a agent (Diphereline, Beaufort Ipsen, France) was initiated at a dosage of 3.75 mg when the B-ultrasonic examination performed on day 2~4 of the menstrual cycle showed that there were no vegetative cysts in bilateral ovaries and blood FSH, LH, E2 and P were in a basic state. 28~35 days after medication, a B-ultrasonic examination and four sexual hormone examinations were performed again. The following criteria were met: E2<110 pmol/L, LH<5 U/L, p<2.86 nmol/L, diameter of bilateral ovarian antral follicles≤5 mm, the thickness of endometrium<5 mm, suggesting that the pituitary down-regulation standard was met. Individualized Gn dosage regimens were developed according to patients’ number of sinus follicles, age, BMI, and response to gonadotropin (Gn, Gonafin, Merck Serono, Switzerland). GnRHa and ovulation-inducing drugs were stopped when there were at least three dominant follicles that had a diameter up to 18mm and according to the levels of LH, E2 and P. HCG 5000~10000 IU or r-HCG (human chorionic gonadotropin, hCG, Ovidrel, Merck, Switzerland) 250 ug was injected intramuscularly that night. Egg retrieval was performed 36 hours after HCG injection.

Gn was applied since day three of the menstrual cycle at an initial dose of 100~225 IU. GnRH-ant (Cetrotide, Merck Serono, Switzerland) was added at a dose of 0.25 mg when the diameter of dominant follicles reaches 12~14 mm or blood E2>551~1468 pmol/L. The time to trigger ovulation was the same as the long follicular phase GnRHa regimen.

After egg retrieval, embryos were cultured in pseriesTM medium to D3 and were evaluated according to ASEBIR embryo evaluation criteria at the cleavage stage.^6^ Embryos rated as Grade-I were high-quality cleavage embryos. The embryos with high scores were transferred. Fresh cycle transfer was canceled and the whole embryo freezing was performed for patients with P≥6 nmol/L on the day of HCG injection, fallopian tube effusion reflux, effusion in the uterine cavity, the thickness of endometrium < 7 mm in the B-ultrasonic examination on the day of HCG injection and ovarian hyperstimulation syndrome. Embryos were transferred on D3, or the remaining cleavage embryos after freezing further underwent blastocyst culture to D5/D6. The formed blastulas were evaluated according to the Gardner blastocyst grading system.^7^ Blastulas of Stage-3 or above with asynchronous trophoblast or inner cell mass rating of C were selected. The cleavage embryos and blastulas were frozen and thawed with the freezing and thawing kit purchased from Japan tazato. The blastulas underwent artificial shrinkage by laser boring before freezing. The embryos were frozen and then resuscitated according to the instructions for the reagent. After resuscitation, the zona pellucida of the cleavage embryos was thinned; and the blastulas of the 3/4 stage were incubated by means of a laser.

### Fresh cycle embryo transfer:

The cleavage embryos with a high D3 score of grade III or above were transferred into the middle and posterior part of the uterine cavity under the guidance of ultrasound after one-section catheterization with a PICC transfer catheter. Thirty five days after the transfer, abdominal ultrasonography showed a gestational sac and fetal heart suggesting clinical pregnancy.

### Resuscitation cycle embryo transfer:

In order to avoid confusion due to embryo quality, the resuscitation cycle embryo transfer was the first resuscitation cycle embryo transfer in patients after whole embryo freezing. Different treatment regimens were adopted according to patients’ own conditions in the resuscitation cycle. At least 1-2 frozen-thawed embryos were transferred on D3 or D5 after the endometrium transformation.

Observation indicators and criteria Embryo utilization rate = number of transferable embryos/number of fertilized eggs × 100%; blastula formation rate = number of blastulas formed/number of blastulas cultured × 100%; clinical pregnancy rate = number of pregnancy cycles of the gestational sac detected under ultrasound × 100%; implantation rate = number of gestational sacs detected under ultrasound/number of embryos transferred × 100%; multiple pregnancy rate = number of cycles of multiple pregnancies/number of pregnancy cycles × 100%; abortion rate = number of abortion cycles/number of clinical pregnancy cycles × 100%.

### Statistical methods:

SPSS23.0 was used for statistical processing and analysis of data. The measurement data conforming to normal distribution were compared by one-way analysis of variance (one-way ANOVA) and were expressed as mean ± standard deviation (*χ̅*±*S*). Measurement data that did not conform to the normal distribution were compared by the rank sum test and were expressed as the median (25%, 75%). LSD-t test was used for comparison between groups. The counting data were expressed as rate (%); and χ^2^ test or Fisher exact test was used for comparison between groups. The *OR* value was calculated by binary Logistic regression analysis. *p<*0.05 was considered statistically significant.

## RESULTS

The differences in patients’ age, the proportion of infertility factors, infertility duration (years), BMI, AMH, basic FSH value and other indicators among groups were not statistically significant (*p>*0.05. Table-I.

**Table-I:** Comparison of Basic Conditions of Patients among Groups [M (P25, P75), (*χ̅*±*S*), %].

Group	Number of cycles	Age (years)	Proportion of primary infertility	Infertility duration (year)	BMI (kg/m2)	AMH (μg/L)	Basic FSH (IU/L)
A	92	30.60±3.52	41.3%	3 (2,5)	22.81±3.13	3.58±1.80	6.54 (5.70,7.59)
B	239	31.29±3.67	47.3%	3 (2,6)	23.68±3.64	3.32±1.79	6.90 (5.92,7.94)
C	55	31.63±4.00	46.7%	3 (2,4)	23.34±3.22	3.52±2.20	6.68 (5.22,8.72)
D	157	31.07±3.37	50.3%	3 (2,5)	23.37±3.72	3.07±1.88	6.94 (5.99,8.36)
*F*/*H*	--	1.560	1.900	4.04	1.364	1.751	4.518
*P*	--	0.198	0.594	0.257	0.253	0.156	0.211

A: long follicular phase regimen and freeze-thawed cycle transfer group; B: long follicular phase regimen and fresh cycle transfer group; C: antagonist regimen and freeze-thawed cycle transfer group; D: antagonist regimen and fresh cycle transfer group.

The number of days of Gn use, the number of oocytes retrieved and the number of available embryos in the long follicular phase regimen and freeze-thawed cycle transfer group were greater than those in the antagonist regimen and freeze-thawed cycle transfer group and the antagonist regimen and fresh cycle transfer group. The differences were statistically significant (*p<*0.05). The total amount of Gn used in the long follicular phase regimen and fresh cycle transfer group was higher than that in the long follicular phase regimen and freeze-thawed cycle transfer group and the antagonist regimen and freeze-thawed cycle transfer group; the embryo utilization rate was higher than that in the long follicular phase regimen and freeze-thawed cycle transfer group and the antagonist regimen and freeze-thawed cycle transfer group; the blastula formation rate was lower than that in the long follicular phase regimen and freeze-thawed cycle transfer group and the antagonist regimen and fresh cycle transfer group. The differences were statistically significant (*p<*0.05). The number of oocytes retrieved in the antagonist regimen and fresh cycle transfer group was lower than that in the other three groups, while the embryo utilization rate was higher than that in the other three groups. The differences were also statistically significant (*p<*0.05). Table-II.

**Table-II:** Comparison of Medication and Laboratory Indicators of Patients among Groups [M(P25,P75), (*χ̅*±*S*), %].

Group	Number of cycles	Total amount of Gn used (IU)	Duration of Gn use (d)	Number of oocytes retrieved	Embryo utilization rate (%)	Blastula formation rate (%)
A	92	2681.90±940.80	12.08±2.40	20 (11,24)	42.94 (584/1360)^b^	39.78 (366/920)
B	239	3005.74±1143.40	12.05±2.52	11 (7,14)^a^	46.55 (952/2045)	33.12 (410/1238)^ad^
C	55	2377.58±808.53^b^	9.83±2.21^ab^	14 (6,24)^a^	42.19 (297/704)^b^	37.52 (185/493)
D	157	2351.22±947.83^b^	10.20±2.59^ab^	8 (5,11)^abc^	53.14 (516/971)^abc^	39.65 (205/517)
*F/H*	--	15.037	35.09	98.62	29.29	12.65
*P*	--	<0.001	<0.001	<0.001	<0.001	0.005

a means p<0.05, compared with Group A; b means p<0.05, compared with Group B; c means p<0.05, compared with Group C; d means p<0.05 compared with group D. A: long follicular phase regimen and freeze-thawed cycle transfer group; B: long follicular phase regimen and fresh cycle transfer group; C: antagonist regimen and freeze-thawed cycle transfer group; D: antagonist regimen and fresh cycle transfer group.

The results of the chi-square test showed that the pregnancy rate and implantation rate in the long follicular phase regimen and freeze-thawed cycle transfer group, the long follicular phase regimen and fresh cycle transfer group and the antagonist regimen and freeze-thawed cycle transfer group were higher than those in the antagonist regimen fresh cycle transfer group. The differences were statistically significant *(p<0.05)*. The differences in the multiple pregnancy rate and abortion rate among groups were not statistically significant (*p>*0.05). Table-III.

**Table-III:** Comparison of Clinical Outcomes of Patients among Groups (%).

Group	Number of cycles	Pregnancy rate (%)	Implantation rate (%)	Multiple pregnancy rate (%)	Abortion rate (%)
A	92	60.87 (56/92)	42.53 (74/174)	32.14 (18/56)	8.93 (5/56)
B	239	53.97 (129/239)	38.23 (177/463)	37.21 (48/129)	3.88 (5/129)
C	55	54.55 (30/55)	38.83 (40/103)	33.33 (10/30)	6.67 (2/30)
D	157	43.31 (68/157)^abc^	27.99 (82/293)^abc^	20.59 (14/68)	8.82 (6/68)
X^2^	--	8.162	12.721	5.171	3.079
P	--	0.043	0.005	0.126	0.375

a means p<0.05, compared with Group A; b means p<0.05, compared with Group B; c means p<0.05, compared with Group C. A: long follicular phase regimen and freeze-thawed cycle transfer group; B: long follicular phase regimen and fresh cycle transfer group; C: antagonist regimen and freeze-thawed cycle transfer group; D: antagonist regimen and fresh cycle transfer group.

In order to reduce the interference of confounding factors, binary Logistic regression analysis was adopted, with continuous variables age, BMI, number of oocytes retrieved and AMH. The binary Logistic regression analysis showed that the increased number of oocytes retrieved is a protective factor for improving the pregnancy rate [OR=1.042,95%CI(1.014-1.071), *p<*0.05], while the advanced age is a risk factor for improving the pregnancy rate [OR=0.948,95%CI(0.904-0.994), *p<*0.05].Table-IV.

**Table-IV:** Identification of influencing factors for clinical pregnancy rate by binary logistic regression analysis.

Item	β	SE	Wald	P	OR	95%CI
Age of female patients	-0.54	0.024	4.863	0.027^a^	0.948	0.904-0.994
AMH	0.007	0.051	0.021	0.884	1.008	0.911-1.114
Number of oocytes retrieved	0.041	0.014	8.807	0.003^a^	1.042	1.014-1.071
BMI of female patients	0.013	0.025	0.253	0.615	1.013	0.964-1.063

a means p<0.05, which was considered statistically significant.

## DISCUSSION

[PJMS1]In this study, the average number of oocytes retrieved in the antagonist regimen and fresh cycle transfer groups was much smaller than that in the other three groups. The binary Logistic regression analysis showed that the increased number is a protective number for a high pregnancy rate. This is close to the conclusion drawn in the study of Junwei Z et al.,^8^ in which the binary Logistic regression analysis of the influencing factors for cumulative pregnancy rate and ovarian hyperstimulation syndrome showed that the increased number of oocytes retrieved is a protective number for cumulative pregnancy rate.

In order to avoid the influence of endometrial factors on pregnancy outcome, the pregnancy rate, implantation rate, abortion rate and other indicators of freeze-thawed cycle transfer by the two regimens after whole embryo freezing were compared in this study. The differences were not statistically significant. Muñoz M et al.^9^ found by observing the development of embryos by time-lapse photography technique that there was no difference in the influence of ovulation-inducing regimens on the quality of embryos. Similarly, the study of Meng W et al.^10^ showed that the antagonist regimen can obtain a similar number of oocytes retrieved, normal cleavage number, number of high-quality embryos and number of available embryos as long follicular phase regimen. However, Qian Y et al.^11^ compared the application of the two regimens in patients with unexplained infertility by age groups and found that the fertility rate, blastula formation rate and high score blastula rate in long follicular phase regimen groups were higher than those in antagonist regimen groups in ≤ 35 or 35-39 age groups.

The implantation process of embryos included the incubation of blastula, identification of blastulas and endometrium, localization, adhesion, invasion and other links. Meanwhile, the endometrium allows the implantation of embryos for a limited period of time, i.e., the receptivity establishment of the endometrium. This period is known as the “window period” of the endometrium. Therefore, it is very important to synchronize endometrium development with embryo development during ovulation induction. A great number of studies^12^,^13^ showed that the antagonist regimen can obtain similar or better pregnancy outcomes to or than the traditional agonist regimen while reducing the dosage, shortening the treatment cycle and reducing the incidence of ovarian hyperstimulation. Nevertheless, some studies^14^ showed that the use of antagonists decreases the expression of HOXA10 in endometrial stromal cells, and causes the preformation of the nucleolar channel system, resulting in the asynchronous development and maturation of endometrium and eggs. Han W et al.^15^ divided the patients who had adopted the long GnRH-a regimen or the antagonist regimen into different age groups and found that the antagonist regimen can reduce the endometrial receptivity in 30-40 years old groups with a normal ovarian response, thus reducing the embryo implantation rate and clinical pregnancy rate. Meanwhile, previous literature^16^ showed that GnRH-a can increase the number of endometrial pinocytosis, increasing the expression of integrin and inhibiting the production of humoral cytokines. In this way, the intrauterine environment is made suitable for embryo implantation. Grow et al.^17^ found by retrospectively analyzing groups with a normal response that, compared with antagonist regimens, long regimens can significantly improve endometrial receptivity and can achieve a higher implantation rate and live birth rate. In this study, the freeze-thawed cycle transfer and fresh cycle transfer by the two ovulation-inducing regimens were compared. The results showed that the pregnancy rate and implantation rate of the fresh cycle transfer by the antagonist regimen were significantly lower than those in the other three groups. Several previous studies^11^,^18^ proved that GnRH-a can increase the endometrial thickness on the day of HCG injection, thus increasing the receptivity of the endometrium and improving the implantation rate of embryos. However, the antagonist regimen decreases the receptivity of the endometrium and reduces the implantation rate of embryos, which is consistent with the result of this study that pregnancy rate and implantation rate in the fresh cycle transfer by long follicular phase regimen and the freeze-thawed cycle transfer by antagonist regimen were significantly higher than those of the fresh cycle transfer by antagonist regimen.

In this study, the data of patients treated with freeze-thawed cycle transfer and fresh cycle transfer by the two regimens were compared for the first time. The influence on asynchronous embryonic development and endometrial development due to the differences in ovulation-inducing regimens was compared among groups.

### Limitations:

The inadequate data of patients treated with freeze-thawed cycle transfer by antagonist regimen may result in deviation in data results. Therefore, it is necessary to further increase patient data.

## CONCLUSIONS

The antagonist regimen reduces dosage, shortens the treatment cycle, and improves the embryo utilization rate. Moreover, the antagonist regimen and freeze-thawed cycle transfer can obtain a similar pregnancy rate and implantation rate to those of the long follicular phase regimen, while the clinical outcome of the antagonist regimen and fresh cycle transfer is significantly lower than that in the other three groups. Therefore, selecting ovulation-inducing regimens and transfer strategies suitable for patients can improve the clinical pregnancy rate.

### Authors’ Contributions:

**PL and JZ** carried out the studies, data collection, drafted the manuscript.

**TL and MG** performed the statistical analysis and participated in its design.

**YW** performed the statistical analysis and participated in its design. Critical Review.

All authors read and approved the final manuscript. They are also responsible and accountable for the accuracy or integrity of the work.
